# TGF-β Signaling in Bone Remodeling and Osteosarcoma Progression

**DOI:** 10.3390/jcm5110096

**Published:** 2016-11-03

**Authors:** Audrey Lamora, Julie Talbot, Mathilde Mullard, Benedicte Brounais-Le Royer, Françoise Redini, Franck Verrecchia

**Affiliations:** 1 INSERM, UMR 957, Equipe Labellisée Ligue contre le Cancer 2012, Faculté de Médecine, 1 rue Gaston Veil, 44035 Nantes cedex, France; audrey.lamora2@gmail.com (A.L.); julie.talbot@curie.fr (J.T.); mathilde.mullard@univ-nantes.fr (M.M.); benedicte.brounais@univ-nantes.fr (B.B.-L.R.); francoise.redini@univ-nantes.fr (F.R.); 2 Laboratoire de Physiopathologie de la Résorption Osseuse et Thérapie des Tumeurs Osseuses Primitives, Université de Nantes, 44000 Nantes, France; 3 INSERM Liliane Bettencourt School, 75014 Paris, France

**Keywords:** bone remodeling, osteosarcoma, TGF-β, primary tumor growth, metastasis

## Abstract

Osteosarcomas are the most prevalent malignant primary bone tumors in children. Despite intensive efforts to improve both chemotherapeutics and surgical management, 40% of all osteosarcoma patients succumb to the disease. Specifically, the clinical outcome for metastatic osteosarcoma remains poor; less than 30% of patients who present metastases will survive five years after initial diagnosis. Treating metastatic osteosarcoma thus remains a challenge. One of the main characteristics of osteosarcomas is their ability to deregulate bone remodelling. The invasion of bone tissue by tumor cells indeed affects the balance between bone resorption and bone formation. This deregulation induces the release of cytokines or growth factors initially trapped in the bone matrix, such as transforming growth factor-β (TGF-β), which in turn promote tumor progression. Over the past years, there has been considerable interest in the TGF-β pathway within the cancer research community. This review discusses the involvement of the TGF-β signalling pathway in osteosarcoma development and in their metastatic progression.

## 1. Introduction

**Figure 1:**
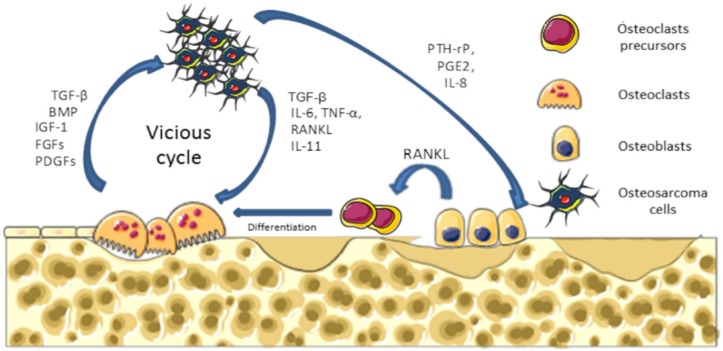
Vicious cycle between primary tumor cell and bone cells. Cancer cells produce soluble factors that activate the osteoclast differentiation and maturation directly or indirectly via osteoblasts. In turn, during bone degradation, osteoclasts allow the release of growth factors stored in the mineralized bone matrix that are able to stimulate tumor growth. TGF-β: transforming growth factor-β.

Osteosarcoma are the most common malignant primary bone tumors affecting children and young adults, with 2–3 cases per million per year [1,2,3]. Osteosarcomas arise from mesenchymal bone-forming cells, and mainly occur in long bone extremities, such as the distal femur, the proximal tibia, or the humerus [4]. Molecular mechanisms underlying osteosarcoma formation are characterized by complex karyotype and multiple genomic alterations [5,6]. Osteosarcomas are pathologies that affect bone remodeling, involving alterations in both osteoblast and osteoclast functions. They are characterized by the direct formation of osteoid matrix by tumor cells, associated with severe osteolytic lesions. To explain these dysregulations of bone cell functions, a vicious cycle between tumor and bone cells has been described during osteosarcoma development (Figure 1). In brief, cancer cells produce soluble factors, such as cytokines (IL-6, IL-11, TNF-α, RANKL, etc.) that activate osteoclastogenesis, leading to bone degradation. Following bone resorption, growth factors trapped in the bone matrix, such as IGF-1 or transforming growth factor-β (TGF-β), are released in the bone microenvironment and stimulate tumor growth [7].

The current treatments include the combination of surgical tumor resection with limb salving and systemic multidrug neoadjuvant and adjuvant chemotherapy [8,9]. Before the introduction of chemotherapy in the early 1980s, amputation was the only therapeutic approach, and survival rates were around 20% at five years. Since then, overall survival had evolved with a five-year survival of about of 70%–75% for localized forms, but still very poor for patients with metastasis at diagnosis [10] or resistant to chemotherapy (approximately 20% at 5 years). New molecular approaches attempt to better understand this disease in order to identify new markers and new therapeutic targets. Among developing treatments, various strategies have been developed, such as targeting of the tumor microenvironment, induction of apoptosis, or inhibition of different signaling pathways [11]. Despite advances in diagnosis and treatments of osteosarcoma, no substantial improvement in survival rate has been achieved over the past few decades, and the mortality rate remains high for high-risk patients [12]. In this context, developing a better understanding of osteosarcoma biology with the aim of identifying new therapeutic targets is a major challenge in order to improve the outcome in osteosarcoma patients with poor prognosis.

## 2. TGF-β Signaling Pathways

The transforming growth factor-β (TGF-β) family of secreted cytokines comprises at least 30 members in humans [13]. Three isoforms—TGF-β1, -β2 and -β3—have been identified in mammals. TGF-βs are secreted as latent precursor molecules requiring activation into a mature form for receptor binding [14]. Once activated, TGF-βs signal from the membrane to the nucleus by binding to two heteromeric cell surface receptors, named type I (TβRI) and type II (TβRII) receptors. Ligand binding induces the assembly of TβRI and TβRII into complexes, within which TβRII phosphorylates and activates TβRI. This phosphorylation event is associated with the activation of TβRI kinase and subsequent downstream signaling [15,16,17,18,19].

**Figure 2:**
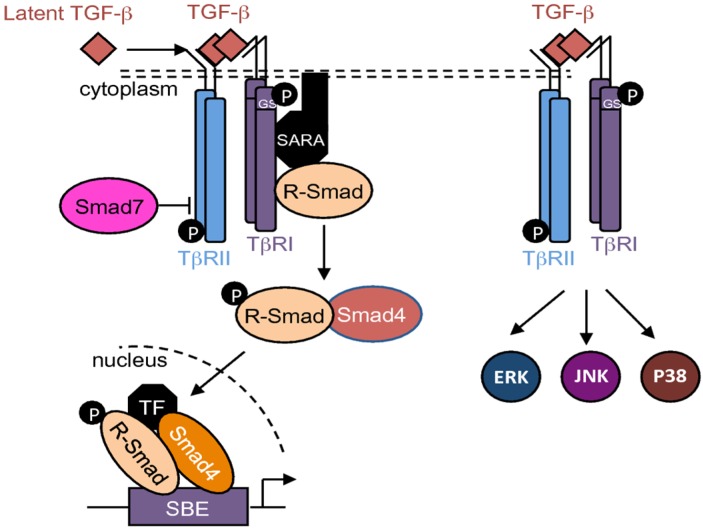
TGF-β signaling pathways. Schematic representation of the canonical and non-canonical TGF-β signaling pathways. R-Smad: receptor-regulated Smad; SBE: Smad-binding element; TF: transcription factor.

TGF-βs thus activate the Smads cascade, known as the canonical TGF-β signaling pathway. Briefly, receptor-regulated Smads (R-Smads), including Smad1, -2, -3, -5, and -8, are phosphorylated and activated by TβRI. Then, R-Smads recruit the common-mediator Smad (co-Smad), Smad4. This protein complex is translocated into the nucleus and regulates target gene expression (Figure 2). At the regulatory DNA binding sequence of genes, the R-Smad/co-Smad complex activates transcription through physical interaction and functional cooperation of DNA-binding Smads with sequence-specific transcription factors [19,20]. The minimal Smad-binding element (SBE) contains four base pairs (5′-AGAC-3′), but binding to other G/C-rich sequences has also been reported [21,22]. TGF-β signalling may be controlled by several inhibitory mechanisms. Among them, Smad7—induced by TGF-β—competes with R-Smads for binding to activated TβRI, and thus inhibits R-Smads phosphorylation and/or recruits E3-ubiquitin ligases to activated TβRI, resulting in receptor degradation [17,23]. Additionally, Smad7 may recruit protein phosphatases to the receptor complex, resulting in its dephosphorylation [24], and thus in its inactivation.

In addition to this canonical pathway, TGF-βs are also able to activate Smad-independent or non-canonical pathways such as PI3K/AKT, ERK1/2, JNK, and p38 cascades (Figure 2) [25,26].

## 3. TGF-β and Bone Remodeling

Bone remodeling mainly depends on the differentiation and activity of two cell lineages: the mesenchymal osteoblastic lineage, and the hematopoietic osteoclastic lineage. At the molecular level, the differentiation and activation processes of these cell lineages are tightly regulated by various cytokines and growth factors, including TGF-βs.

Although TGF-β1 is the most abundant in bone [27,28], the three mammalian isoforms (TGF-β1, -β2, and -β3) are found in bone, particularly expressed by the perichondrium, the periosteum, and the epiphyseal growth plate [29]. Latent precursor molecules of TGF-β1 are in part synthetized by osteoblasts, deposited in the bone matrix, and activated by acids and matrix metalloproteinases secreted from osteoclasts [30].

The role of TGF-β1 in skeleton development, and specifically during bone remodeling is complex. Regarding the mesenchymal osteoblastic lineage, TGF-β1 favors bone formation by stimulating the proliferation and migration of mesenchymal stem cells during the early stages of osteoblastogenesis [30,31]. In contrast, during the late stages of osteoblastogenesis, TGF-β1 inhibits the differentiation of mesenchymal stem cells into osteoblasts and the mineralization of mature osteoblasts in culture [32,33]. Regarding the hematopoietic osteoclastic lineage, TGF-β1 affects bone resorption in a dose-dependent manner [29]. Low concentrations of TGF-β1 stimulate the migration of osteoclast precursors to the bone resorption site, and their differentiation into mature osteoclasts. In contrast, at high doses, TGF-β1 inhibits the migration of osteoclast precursors and their differentiation through the modulation of RANKL and OPG expression by osteoblasts [34,35]. In vivo experiments indicate that TGF-βs favor bone resorption and destruction [36,37,38].

## 4. TGF-β and Cancer

TGF-βs are able to regulate tumor initiation, progression, and metastatic development. It is widely accepted that TGF-βs act as both tumor suppressors and tumor promoters, depending on the cancer type and tumor development timing [39,40,41,42,43,44]. 

During the early stage of tumor development, TGF-β1 acts as a tumor suppressor mainly by its ability to inhibit the proliferation of epithelial cells. TGF-β1 can cause G1 cell cycle arrest by inducing the expression of CDK inhibitors such as p21^Cip1^ and p15^lnk4b^, and/or reducing the expression of proliferative drivers such as c-Myc and ID [45,46,47]. Other tumor-suppressing properties have been correlated to the ability of TGF-βs to induce the apoptosis [48] or senescence of cancer cells [49]. In this context, alterations of the TGF-β cascade have been associated with many cancers [40,50,51]. For example, Smad4 gene mutations have been identified in most pancreatic [52] and colorectal [53] cancers, and in a lesser proportion in other cancers, such as hepatocellular, ovarian, intestinal, and lung carcinomas [40]. Mutations of TβRI and TβRII have also been identified in many cancers; TBRII has been associated with colon, gastric, pancreatic, lung, and brain tumors, for example [50,54,55,56], and mutations of the TβRI gene have been identified in ovarian tumors [51].

During the last decades, studies of TGF-β expression in epithelial cancers have correlated the levels of TGF-β with the metastatic potential of many tumors, such as breast, colon, and prostate [57,58,59], suggesting a role of TGF-β in tumor progression. It is now well accepted that TGF-βs act as tumor promoters during the late stages of carcinogenesis, by their ability to induce epithelial–mesenchymal transition (EMT), to stimulate angiogenesis, and to favor immune evasion.

EMT—characterized by the loss of E-cadherin, the expression of mesenchymal cytoskeleton proteins such as vimentin and fibronectin, and the expression of transcription factors such as Snail, Slug, Twist, and FoxC3—is induced by TGF-β in many cancer cells [41,60,61,62,63]. The loss of E-cadherin has been associated with Smad-dependent and Smad-independent signaling pathways [64,65,66]. Interestingly, several other signaling cascades, such as the Wnt, Hippo, and Sonic Hedgehog (SHH) cascades cooperate with the Smad cascade to regulate EMT in cancer cells [63]. During the EMT process, the epithelial cells trans-differentiate into mesenchymal cells able to migrate through the extracellular matrix and form metastases at distant secondary sites [67,68]. In this context, TGF-β1 is able to stimulate the expression and activity of MMP-2 and MMP-9, two matrix metalloproteinases implicated in the ability of cancer cells to invade surrounding tissue [69,70]. EMT seems to be a transient and reversible process during carcinogenesis, allowing the promotion of cancer cells’ intravasation into the blood or lymph systems; however, the phenotype of the tumor cells at the metastatic site seems to be mainly determined by the stromal site itself rather than the innate properties of the cancer cells [68].

Tumor-associated angiogenesis also plays a crucial role during tumor progression [71]. This process favors the formation of new blood vessels, allowing the supply of nutrients and providing an entry point for the metastatic cells [72]. As an example, high levels of TGF-β1 mRNA in breast cancers are associated with an increase in the density of blood vessels [73]. Other studies suggest that the level of TGF-β1 in the circulating plasma is associated with the induction of tumor angiogenesis [59,74,75,76]. It seems that TGF-β stimulates angiogenesis in part by stimulating the expression of vascular endothelial growth factor (VEGF) and connective tissue growth factor (CTGF) [40].

A third crucial step in cancer progression is the selective suppression of the immune system. TGF-βs produced by several immune cells, such as macrophages, dendritic cells, NK cells, B cells, and T cells play a crucial role in the suppression of the immune system, as demonstrated by the autoimmunity developed in TGF-β1 null mice [43,77].

## 5. TGF-β and Osteosarcoma

In contrast with the dual effects of TGF-βs on carcinoma progression, TGF-βs seem to mainly have a pro-tumoral effect on sarcoma specifically in osteosarcoma.

The expression of TGF-βs is increased in the sera of patients with osteosarcoma compared to the sera of healthy donors [38]. Interestingly, this increase of TGF-βs production is associated with the presence of metastases in lung or in other sites [78,79], and is correlated with high-grade osteosarcoma and a lack of osteosarcoma response to chemotherapy [80,81].

In vitro experiments have demonstrated the pro-migratory effect of TGF-β1 on several osteosarcoma cell lines [38,82,83,84], this effect being associated with the ability of TGF-βs to promote an EMT-like phenomena [85]. TGF-β1 also exerts pro-angiogenic properties in osteosarcoma [86,87]. In addition, the anti-tumor effects of an anti-TGF-β antibody combined with dendritic cells has been associated with the restoration of the immune response in osteosarcoma [88].

More recently, using molecular (over-expression of the inhibitor Smad, Smad7) and pharmacological (SD-208 and/or halofuginone) approaches, we clearly demonstrated that TGF-βs affect osteosarcoma tumor growth and lung metastatic development [38,89]. Of note, SD-208 is a chemical inhibitor of TβRI, and halofuginone is an alkaloid known for its inhibitory properties on the TGF-β signaling pathway [90]. Using a xenograft murine model of osteosarcoma, we specifically demonstrated that Smad7 overexpression slows primary tumor growth. Interestingly, this effect seems to involve the bone tumor microenvironment rather than the tumor cells directly. Using micro-computed tomography analysis, we indeed demonstrated that Smad7 inhibits tumor-associated bone destruction by both promoting ectopic bone formation and preventing trabecular bone osteolysis. Our hypothesis is that blocking the TGF-β cascade in tumor cells inhibits the expression of TGF-β target genes involved in the establishment of the vicious cycle between tumor cells and bone cells. In this context, we demonstrated that Smad7 overexpression in osteosarcoma cells blocks their ability to produce RANKL, a cytokine that plays a central role in osteoclast differentiation and activation [91]. In addition, we demonstrated that blocking the TGF-β cascade in tumor cells inhibits the expression of TGF-β target genes, such as IL-11, CXCR4, and osteopontin, known to enhance bone metastasis formation from breast cancer cells or melanoma [92,93,94,95]. Of note, in contrast to Smad7, the effects of halofuginone appear to be mainly due to its pro-apoptotic properties in osteosarcoma, regardless of its ability to inhibit the TGF-β signaling pathway [89]. The role of the non-canonical TGF-β signaling pathway in osteosarcoma progression is poorly documented. Although we have not observed an effect of Smad7 on the ability of TGF-β to stimulate the MAPK pathway in osteosarcoma cells (suggesting a crucial role of the TGF-β/Smad cascade in osteosarcoma progression), the role of TGF-β/MAPK pathways cannot be ruled out.

**Figure 3:**
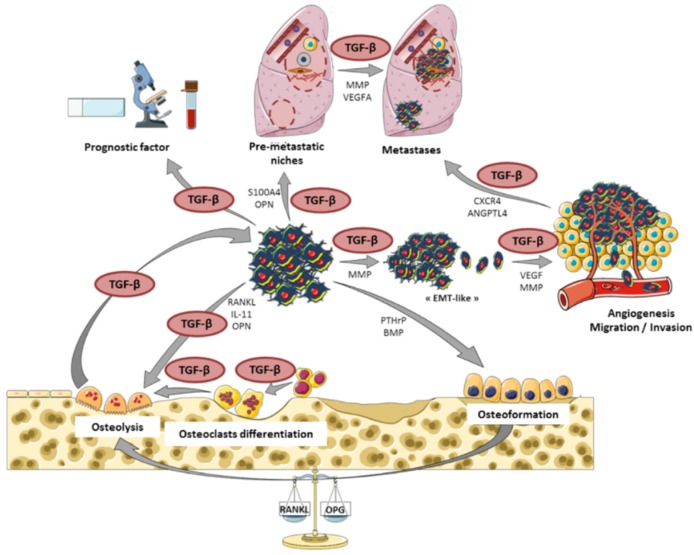
The central role of TGF-β in osteosarcoma tumor and metastases development. Roles of TGF-β as a main player in the vicious cycle between osteosarcoma cells and the bone tumor microenvironment, thus contributing to tumor development and lung metastases dissemination. EMT: epithelial–mesenchymal transition; MMP: matrix metalloproteinase; VEGF: vascular endothelial growth factor.

Finally, we showed that Smad7, SD-208, and halofuginone strongly affect the ability of the primary bone tumor to develop lung metastases, mainly by their ability to block the capacity of TGF-β1 to stimulate osteosarcoma migration and invasion, as previously described in the context of melanoma bone metastasis [93,94,95]. The roles of TGF-β in the progression of osteosarcoma and the development of lung metastases are summarized in Figure 3.

## 6. TGF-β Cascade Blockers in Cancer Clinical Trials 

During the last decade, numerous strategies against TGF-β signaling have been used in preclinical or clinical applications, especially in end-stage cancer, including anti-ligand antisense oligonucleotides, antibodies that target ligands or receptors, and drugs against TGF-β receptor kinases (reviewed in [40,96]). Anti-TGF-β2 antisense strategies have thus been developed. For example, Trabedersen (AP12009)—a synthetic 18-mer phosphorothioate antisense oligonucleotide able to bind human TGF-β2 mRNA—has been successfully used in clinical trials for oncological applications [40,96], such as glioblastoma [97,98]. Strategies using monoclonal antibodies have also been developed. For example, GC-1008 (Fresolimumab, a humanized mAB against TGF-β) was developed and tested in Phase I/II clinical trials on patients with advanced malignant melanoma or renal carcinoma [99]. Many chemical compounds able to block the transduction of TGF-β signal, such as inhibitors of TβRI or TβRII (SB-431542, LY2109761, or LY2157299, etc.) have been developed in preclinical models. Among them, only one (LY2157299) was recently tested in clinical trial on patients with Grade IV glioma [100]. 

## 7. Conclusions and Perspective

In conclusion, blocking TGF-β signaling may represent a promising therapeutic approach to treat osteosarcoma patients. However, despite the existence of many tools allowing us to block TGF-β signalling pathways, such as neutralizing antibodies, soluble TGF-β receptors, or receptor kinase inhibitors, the lack of spectacular success in clinical trials reinforces the need to continue research on this TGF-β signaling pathway, specifically on the crosstalk between this pathway and others implicated in osteosarcoma tumor progression, such as Wnt, Hippo, or SHH cascades.
